# Projecting COVID-19 intensive care admissions for policy advice, the Netherlands, February 2020 to January 2021

**DOI:** 10.2807/1560-7917.ES.2024.29.10.2300336

**Published:** 2024-03-07

**Authors:** Don Klinkenberg, Jantien Backer, Nicolette de Keizer, Jacco Wallinga

**Affiliations:** 1 National Institute for Public Health and the Environment, Bilthoven, The Netherlands; 2 National Intensive Care Evaluation (NICE) Foundation, Amsterdam, The Netherlands; 3 Department of Medical Informatics, Amsterdam UMC, Amsterdam Public Health Research Institute, Amsterdam, The Netherlands; 4 Leiden University Medical Centre, Leiden, The Netherlands

**Keywords:** SARS-CoV-2, simulation model, mathematical model, forecasting, epidemic, policy advice

## Abstract

**Background:**

Model projections of coronavirus disease 2019 (COVID-19) incidence help policymakers about decisions to implement or lift control measures. During the pandemic, policymakers in the Netherlands were informed on a weekly basis with short-term projections of COVID-19 intensive care unit (ICU) admissions.

**Aim:**

We aimed at developing a model on ICU admissions and updating a procedure for informing policymakers.

**Method:**

The projections were produced using an age-structured transmission model. A consistent, incremental update procedure integrating all new surveillance and hospital data was conducted weekly. First, up-to-date estimates for most parameter values were obtained through re-analysis of all data sources. Then, estimates were made for changes in the age-specific contact rates in response to policy changes. Finally, a piecewise constant transmission rate was estimated by fitting the model to reported daily ICU admissions, with a changepoint analysis guided by Akaike's Information Criterion.

**Results:**

The model and update procedure allowed us to make weekly projections. Most 3-week prediction intervals were accurate in covering the later observed numbers of ICU admissions. When projections were too high in March and August 2020 or too low in November 2020, the estimated effectiveness of the policy changes was adequately adapted in the changepoint analysis based on the natural accumulation of incoming data.

**Conclusion:**

The model incorporates basic epidemiological principles and most model parameters were estimated per data source. Therefore, it had potential to be adapted to a more complex epidemiological situation with the rise of new variants and the start of vaccination.

**Key public health message**

**What did you want to address in this study and why?**

During the COVID-19 pandemic there was a real risk that healthcare demand would exceed the available capacity. Policymakers needed accurate projections of the daily number of hospitalisations and intensive care unit (ICU) admissions to decide on control measures. We used a model with real-time data for weekly projections for policymakers in the Netherlands. The actual ICU admissions matched the projections remarkably well.

**What have we learnt from this study?**

The model projected too many ICU admissions at the peak of the first pandemic wave (spring 2020), when the behaviour change was more drastic than required by the measures; projections were too low during the second pandemic wave (autumn 2020), caused by the seasonality in SARS-CoV-2 transmission. In both cases, the quality of the projections improved quickly when the model was updated with incoming data.

**What are the implications of your findings for public health?**

We show that integrating real-time data in a transmission model has potential for timely projections that are informative to policymakers. As the update procedure consisted of separate adaptable steps, from data analysis to projections, it was modified to address more complex epidemiological situations, with different vaccines and with emerging variants, in the later stages of the pandemic.

## Introduction

Since early 2020, the coronavirus disease 2019 (COVID-19) pandemic has had a severe impact on society. To mitigate the primary impact due to infection with the with severe acute respiratory syndrome coronavirus 2 (SARS-CoV-2), including the large burden on healthcare systems affecting both quantity and quality of care beyond COVID-19, public health control measures were taken that seriously affected everyday life, economically as well as socially [1,2]. A great responsibility lay with the policymakers who had to weigh these factors into a balanced control policy, despite all uncertainties about both the positive and negative effects of the control measures.

An important source of information guiding the decision to implement or lift a control measure, was the input from epidemic models. In the Netherlands, the National Institute for Public Health and the Environment (RIVM) was responsible for modelling to inform policymakers. Results were presented weekly to the Outbreak Management Team, the official medical advisory body to the government. Models were developed for various objectives, such as to advise on the introduction or optimisation of particular control measures, including contact tracing and vaccination [3,4] and to assess the current state of the epidemic, by nowcasting and estimating the effective reproduction number [5,6].

To assess the effect of control measures in place and to allow timely decisions regarding modifications of control, there was a need for short-term projections (up to 3 weeks) of admissions to Intensive Care Units (ICU). Many forecasting models have been used worldwide, which make use of mechanistic transmission models or statistical techniques such as time series analyses, filtering methods and machine learning [5,7-14]. In the Netherlands, the National Centre for Patient Exchange (LCPS), the body responsible for hospital capacity planning and patient relocations, produced weekly short-term forecasts for hospitals. These were based on extrapolation of the current growth rate in hospital admissions [15]. The Netherlands Organisation for Applied Scientific Research (TNO) developed a transmission model that was briefly used for forecasts [16], using publicly available data on ICU admissions in an ensemble smoother fitting algorithm. Both the LCPS and TNO models were not designed to project the effect of recent changes in control measures.

To provide policymakers with short-term projections of new admissions, resulting healthcare demand and the effects of control measures, we developed a dynamic transmission model for the spread of SARS-CoV-2 in the Dutch population. The model was primarily used for projections of hospital and ICU admissions on a 3-week time horizon, and a modelling procedure was designed to minimise the need for arbitrary decisions about the impact of control measures. Here we present the model and the procedure for integration of the most recent data and recent changes in control measures. Although the procedure was followed for the entire period up to March 2022 with major contact-restricting control policy in place, in this article we present the model used between 28 March 2020 and 6 January 2021 before vaccination and the rise of new variants.

## Methods

The model incrementally combined incoming data with a mechanistic description of recent changes in control measures to make projections for the course of the epidemic on a 3-week time horizon. On the day of analysis, the most recent data were collected to parameterise and fit the model.

### Data

#### Population

We used the population size and age structure of the Netherlands in 2020, published by the Statistics the Netherlands (CBS), in age groups of 10 years: 0–9, 10–19, 20–29, 30–39, 40–49, 50–59, 60–69, 70–79, ≥ 80 years (www.opendata.cbs.nl).

#### Contacts

In 2016–2017, a nationwide study including a contact survey was conducted in the Netherlands [17]. In this contact survey, called Pienter study, participants aged 0–89 years (or the parents in case of child participants) filled out a contact diary over 1 day, in which they registered all their contacts specified by age and setting: work, school, home, leisure, transport or other.

In 2020, an international collaboration started collecting contact survey data in several European countries (CoMix) [18]. We used the data of the first survey conducted in the Netherlands in early April 2020 [19].

#### Serology

In April 2020, the first serological survey in the general population for antibodies against SARS-CoV-2 was carried out in the Netherlands, followed by a second survey in June 2020 [20,21]. These surveys, called PiCo, provided estimated seroprevalences for the age groups described above, for the mean days of sample collection of 6 April and 15 June.

#### Case notifications

Detection of SARS-CoV-2 by PCR is notifiable and all cases were registered in the Osiris database [22]. Relevant variables for this study were: age, date of symptom onset, date of hospital admission and information on the potential infector. Following initial notification, records were not always updated, resulting in incomplete hospitalisation records in this database, especially after June 2020, when community-wide testing became available for everyone with COVID-19-like symptoms.

#### Admissions to hospitals and intensive care units

The National Intensive Care Evaluation (NICE) registered all COVID-19 hospital admissions in the Netherlands [23]. Records consisted of dates of admission and discharge or death for every patient in every ward, with pseudonymised patient identifiers allowing the linkage of potential multiple records for a single patient. As real-time data were used, the duration of stay was censored for many patients, especially during periods of high occupancy.

#### Ownership and privacy

Most datasets contained personal information and were therefore privacy sensitive, only CBS is open data. Pienter, CoMix, and PiCo data were collected and owned by RIVM. Osiris data were collected by the Dutch municipal health services and reported to RIVM. Data on hospital admissions were processed by NICE Research and Support under supervision of the NICE foundation. For research purposes, RIVM was allowed to use these data sources. Anonymised case data and aggregated data were made available for public use on the RIVM website (https://data.rivm.nl/covid-19/). For the weekly data analysis, the most recent individual-based data were always used.

### Model

**Figure 1:**
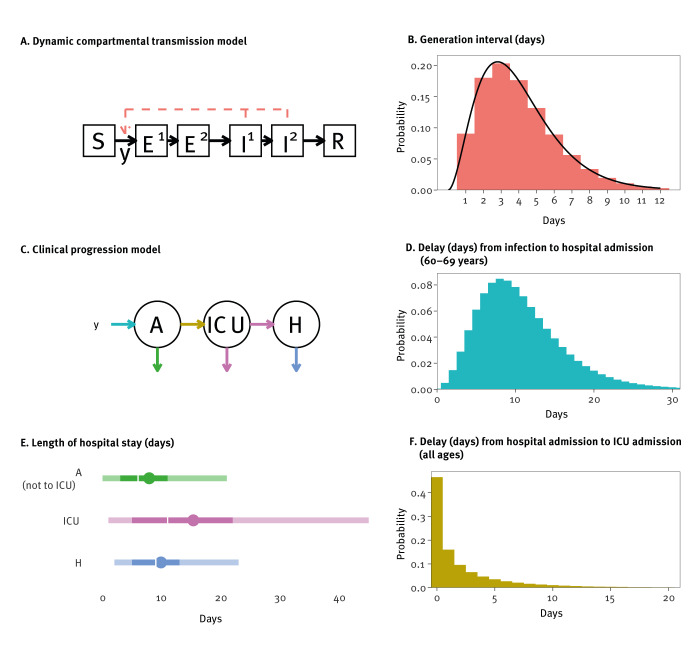
Simulation model of coronavirus disease 2019, by age group, the Netherlands, 2020–2021

We used a deterministic age-structured susceptible-exposed-exposed-infected-infected-removed/recovered (SEEIIR) compartmental model for transmission of SARS-CoV-2 in the Netherlands (Figure 1A), described with ordinary differential equations (ODEs), for nine age groups (0–9, 10–19, 20–29, 30–39, 40–49, 50–59, 60–69, 70–79, ≥ 80 years). In this model structure, an individual starts in the *S* (susceptible) compartment, enters the *E*^1^ (latently infected) compartment by infection at rate *λ*(*t*) and then moves through each of the *E*^2^, *I*^1^, and *I*^2^ compartments into *R* (immune or dead), with each transition made at the same rate *γ*. Cases were not infectious in the *E*^1^, *E*^2^ and *R* compartments and were equally infectious in both *I* compartments. This structure served to obtain the desired generation interval distribution (Figure 1B). The model was initialised on 12 February 2020 with *y*_0_/4 individuals in each age group in each of the four compartments *E^1^*, *E^2^*, *I^1^* and *I^2^*. The infection incidence *y_i_(t)* in age group *i*, i.e. the daily rate of new infections of susceptible individuals having contacts with infected individuals in both infectious classes (*I* = *I*^1^ + *I*^2^), was modelled as


$$\begin{array}{l} \text{Formula 1: }\\ y_{i}\left(t\right)=\lambda_{i}\left(t\right)S_{i}\left(t\right)\\ \lambda_{i}\left(t\right)=\beta \left(t\right)\sigma_{i}^{S}\sum_{j}c_{ij}\left(t\right)\sigma_{j}^{I}I_{j}\left(t\right)/N \end{array}$$

A: admission to a general ward of a hospital; E: exposed individual; H: hospital stay (general ward); I: infected and/or infectious individuals; ICU: intensive care unit; R: immune or dead individual; S: susceptible individual; SEEIIR: susceptible-exposed-exposed-infected/infectious-infected/infectious-removed/recovered; y: incidence of infection.

Panel A. Dynamic age-stratified SEEIIR compartmental transmission model, with incidence of infection *y* from susceptible (*S*) to exposed (first stage) (*E*^1^) and equal fixed rates between all compartments from exposed (first stage to exposed (second stage) to both infected and infectious (*I*^1^ and *I*^2^), and finally to either immune or dead (*R*). Incidence results from contacts of susceptibles with infected and infectious persons.

Panel B. Continuous generation interval distribution resulting from the SEEIIR structure of the transmission model (line) and the discretised version *g*(*τ*) used for the discrete-time model (histogram).

Panel C. Clinical progression model from infection incidence to hospital admission (general ward), transfer to the ICU, and transfer back to a general hospital ward, with outflow because of discharge or death.

Panel D. Delay distribution from infection to hospital admission (general ward).

Panel E. Length-of-stay distributions on the general ward (patients not going to ICU), on the ICU and on the general ward (after discharge from the ICU). Points are means, white gaps are medians, solid colours are 50% range, transparent colours are 90% ranges.

Panel F. Delay distribution from hospital admission to ICU admission.

Panels D-F situation from 6 January 2021, with colours matching the arrows in C.

This assumes homogeneous mixing within age groups. The time-varying force of infection *λ_i_*(*t*) is the fundamental descriptor of the transmission model and combines the underlying components infectivity, susceptibility and contact rates between individuals of all age classes, as well as how these contact rates changed due to control measures, behaviour or by other means (see next section for parameter estimation). The components of *λ_i_*(*t*) consist of:

- the population size *N*, with proportion of the population *x_i_* in age group *i* to determine the initial values *S_i_*(0) = *x_i_ N* – *y*_0_
- the number of infected people in age group *j*, *I_j_*
- the relative susceptibility of age group *i*, *σ_i_^S^*, and relative infectivity of age group *j*, *σ_j_^I^*
- the rate by which each individual in age group *i* makes contacts with individuals of age group *j* (if all individuals would be of type *j*), *c_ij_*(*t*). This rate is an element of the contact matrix **C**(*t*) and is stepwise constant, depending on control measures in periods of the epidemic *T* ending at transition times *u_T_* (*u*_0_ is before the pandemic). Given the contact matrices **C***_T_*, and the transition times *u_T_*,
  $$C\left(t\right)=C_{T,u_{T-1}<t\leq u_{T}}$$
- the transmissibility parameter, *β*(*t*). This rate is stepwise constant with changepoints at some of the transition times *u_T_*, which reflect changes in transmissibility that are not covered by changes in the contact matrices:
  $$\beta \left(t\right)=\beta_{T,u_{T-1}<t\leq u_{T}}$$

The basic reproduction number *R*_0_ of this model, defined as the mean number of secondary cases per primary case in a susceptible population, is equal to the largest eigenvalue of the next-generation matrix [24]


$$\begin{array}{l} \text{Formula 2:}\\ M_{1}\left(0\right)=\frac{2\beta \left(0\right)}{\gamma }{\left(\sigma^{I}\right)}^{\text{T}}C_{1}\left(\sigma^{S}\circ x\right) \end{array}$$

in which ◦ indicates element-wise multiplication. The reduction in contact rates due to control measures results in a relative transmission rate *ϕ_T_*(*t*), which is determined by replacing *β*(0) and **C**_1_ by *β*(*t*) and **C**(*t*), and calculating the ratio of largest eigenvalues


$$\begin{array}{l} \text{Formula 3:}\\ \phi_{T}\left(t\right)=\rho \left(M_{T}\left(t\right)\right)/\rho \left(M_{1}\left(0\right)\right) \end{array}$$

To decrease simulation time, as of 25 November 2020 this continuous-time version of the model was not used anymore and replaced (for the entire epidemic) by a discrete-time version with time steps of one day, with *I_j_*(*t*) described in terms of earlier incidence up to 12 days previously (renewal equation [24]) and generation interval distribution *g*(*τ*) (Figure 1B):


$$\begin{array}{l} \text{Formula 4: }\\ I_{j}\left(t\right)=\sum_{\tau =1}^{12}g\left(\tau \right)y_{i}\left(t-\tau \right) \end{array}$$

This model was initialised with incidence *y*_0_/12 in each age group on each day between 1 and 12 February 2020.

To simulate the daily numbers of hospital and ICU admissions and the daily numbers of discharges and deaths, the daily infection incidence *y_i_*(*t*) was used as input for the clinical progression model (Figure 1C). In this model, the expected numbers of hospital and ICU admissions and occupancy were calculated, by using age-specific time-varying probabilities and delay distributions (Figure 1D-F). For instance, the expected number of ICU admissions at time *t* for age group *i* was:


$$\begin{array}{l} \text{Formula 5:}\\ z_{i}\left(t\right)=\sum_{\tau =0}^{t}\sum_{\tau '=\tau }^{t}\sum_{\tau ''=\tau '}^{t}y_{i}\left(\tau \right)p_{i}^{A}\left(\tau ''\right)p_{i}^{ICU}\left(\tau ''\right)d^{S}\left(\tau '-\tau \right)d_{i}^{A}\left(\tau ''-\tau '\right)d^{ICU}\left(t-\tau ''\right) \end{array}$$

This is a convolution of three delay distributions: the delay from infection to symptom onset, from symptom onset to hospital admission and from hospital admission to ICU admission. Here, *p_i_^A^*(∙) and *p_i_^ICU^*(∙) were the respective probabilities of hospital admission and transfer to the ICU, stratified by age and as a function of date of hospitalisation; and *d^S^*(∙), *d_i_^A^*(∙), and *d^ICU^*(∙) were the three delay distributions.

### Estimation of parameters and distributions

#### Step 1: parameters and distributions (excluding contact rates and transmissibility)

Each weekly model analysis started with the collection of all parameter values other than the contact matrices **C***_T_*, initial state *y*_0_ and transmissibility parameters *β_T_* (Table). The analyses were adapted, when necessary, throughout the year, when additional data sources became available or if parameter values appeared to change over time. We describe a complete analysis as carried out on 6 January 2021 in the Supplement.

**Table:** Overview of parameters used for modelling of transmission of severe acute respiratory syndrome coronavirus 2, the Netherlands, 2020–2021 (population 17.3 million)

Parameter	Data source	Age group (years)								
		0–9	10–19	20–29	30–39	40–49	50–59	60–69	70–79	≥ 80
Age distribution (proportions)	CBS	0.103	0.116	0.127	0.122	0.131	0.145	0.121	0.088	0.046
Incubation period distribution	[31,32]	Weibull (shape = 2.1, mean = 5.0)								
Generation interval distribution	[33,34]	Figure 1B								
Susceptibility and infectivity	PiCo and Pienter	1^a^	3.05	5.75	3.54	3.71	4.36	5.69	5.32	7.21
Probability of hospital admission after infection^b^	PiCo and NICE	0.003	0.000	0.001	0.004	0.008	0.017	0.025	0.049	0.046
Mean delay from symptom onset to hospital admission(days) ^b^	Osiris	2.3	5.5	5.1	5.7	6.5	5.9	5.7	5.1	4.3
Probability of transfer to ICU from a hospital ward^b^	NICE	0	0.062	0.096	0.110	0.164	0.202	0.244	0.196	0.035
Probability of transfer from ICU to a hospital ward^b^	NICE	0.866	0.866	0.866	0.866	0.866	0.829	0.720	0.573	0.555
Delay and length-of-stay distributions	NICE	Figure 1D-F								

CBS:  Statistics the Netherlands; NICE:  the National Intensive Care Evaluation, data on hospital admissions; Osiris: Dutch database on notifiable diseases; PiCo:  serological survey in the general population for antibodies against SARS-CoV-2; Pienter:  a nationwide study including a contact survey [17].

^a^ Reference group.

^b^ Time-varying parameters, values of 6 January 2021.

#### Step 2: the contact matrices

**Figure 2:**
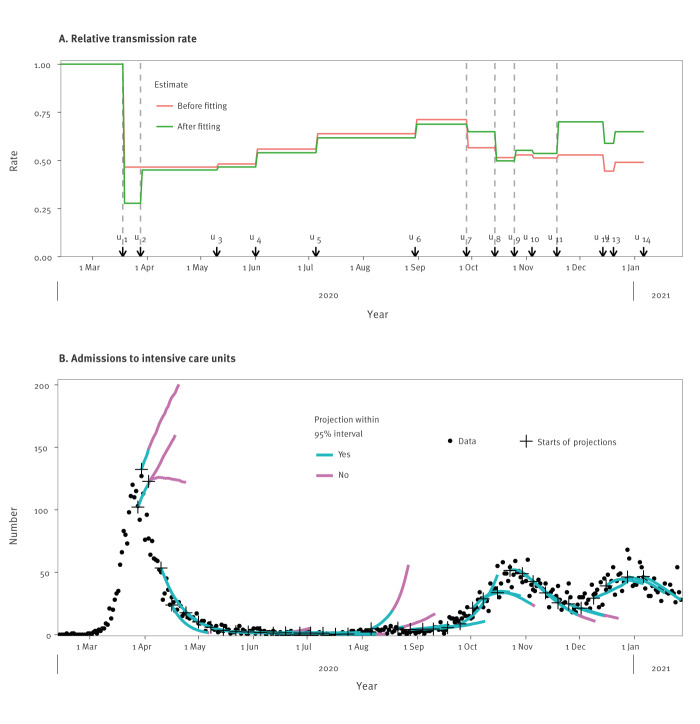
Severe acute respiratory syndrome coronavirus 2 transmission rates and admissions to intensive care units of individuals with COVID-19, the Netherlands, 2020–2021

In the model, contact matrices **C***_T_* describe how different age groups interact with each other in consecutive periods *T* of the pandemic during which different sets of control measures were in place. Each matrix was used up to transition time *u_T_*, the time of policy change (Figure 2A). The sets of control measures and associated reductions in contact rates can be found in the Supplement. An exception was made for the rapid sequential deployment of control measures mid-March 2020, with associated adaptations in contact behaviour. For March 2020, we assumed the existence of two transition times, with the same contact matrix after the first and second transition. The dates of these transitions, *u*_1_ and *u*_2_, were estimated in step 3 (below). These dates do not necessarily correspond to the actual days when major policy changes were implemented.

*u_T_*: transition times.

A. Transmissibility relative to pre-pandemic transmissibility potential, before and after fitting to daily admissions to intensive care units using formulas 2 and 3. The arrows indicate transition times between contact matrices; the dotted vertical lines indicate the changepoints in stepwise transmissibility.

B. All 3-week projections (n = 40), starting on dates marked with crosses, of daily admissions to intensive care units until 6 January 2021, with the blue colour indicating when actual admissions which are shown as dots were within the 95% interval.

The analyses in Panel A were performed on 6 January 2021.

The matrices were based on contact data collected for the Pienter study in 2017, before COVID-19 [17]. In this contact survey, participants recorded their contacts over the course of a day in different settings: home, school, work, leisure (activities such as concerts, physical exercise/sports, seeing friends), transport and rest. Measures against COVID-19 can affect these contacts; how much, in which setting and in which age group will depend on the specific measure. Estimating the reduction in contacts, something that cannot be measured, was done by treating it as a Fermi problem, i.e. by breaking it down many smaller estimation problems which reduces the expected error of the problem as a whole [25,26]. For each set of COVID-19 measures, two researchers independently predicted how the measures would affect the contacts of each age group in each setting, while ensuring consistency with previous sets of measures. This yielded the Fermi estimates, i.e. consensus estimates for the contact rate reductions with an uncertainty range, which were used to subsample the Pienter data and estimate the contact matrices **C***_T_* to ensure the reciprocity of the contacts between age groups [27]. By uniformly sampling from the uncertainty ranges of the Fermi estimates, 200 contact matrices were obtained to describe the effect of a given set of COVID-19 control measures. On 28 May 2020, new estimates were made for all sets of control measures, to match the observations of the first CoMix contact study [18,19] and new matrices were created.

#### Step 3: the transmissibility parameter

The transmissibility parameters *β_T_* and matrix transition times *u_T_* were required for the rate of transmission *β*(*t*). Together with the initial state *y*_0_, all *β_T_*, *u*_1_ and *u*_2_ were estimated by fitting the model to the ICU admission data, conditional on all parameter values as inferred in steps 1 and 2, and all transition times *u_T_* for *T* > 2. We used the optim function in R version 3.6.0 [28] to maximise the log-likelihood, with *T*_max_ stepwise constant transmissibilities in *β*(*t*):


$$\begin{array}{l} \text{Formula 6:}\\ l\left(y\left(0\right),u_{1},u_{2},\beta_{1},...,\beta_{T_{\max}}\left|Z\left(\right),u_{3},...,u_{T_{\max}},\theta ,C_{1},...,C_{T_{\max}}\right.\right)=\\ \sum_{t}Z\left(t\right)\log\left(p_{obs}\left(t_{analysis}-t\right)\sum_{i}z_{i}\left(t;y\left(0\right),\beta_{1},...,\beta_{T_{\max}},\theta ,u_{1},...u_{T_{\max}},C_{1},...,C_{T_{\max}}\right)\right) \end{array}$$

Here, *Z*(*t*) are the observed daily ICU admissions, which were assumed to follow a Poisson distribution, *θ* all parameters estimated in step 1, **C***_T_* the means of all sets of 200 sampled contact matrices, *p_obs_*(*t_analysis_* - *t*) the probability of reporting at the day of analysis, and *z_i_*(*t*;…) the simulated numbers of admissions.

At each later transition time *u_T_*, the change in contact matrices from **C***_T_* to **C***_T_* _+ 1_ reflected the anticipated changes in contact behaviour caused by the policy change at that moment, as estimated in step 2. If the changes in behaviour were correctly reflected in the matrices, it could be assumed that *β_T_* _+ 1_ = *β_T_*, thus reducing the number of model parameters. As a consequence, the stepwise constant transmissibility parameter *β*(*t*) would have fewer changepoints (where *β_T_* _+ 1_ ≠ *β_T_*) than there are transition times. We fitted models with changepoints at different subsets of transition times *u_T_* and selected the set with the lowest value of the Akaike's Information Criterion (AIC) [29]. From the final selected model, point estimates were obtained with a covariance matrix for all stepwise constant transmissibilities and the initial number of infected persons, calculated from the Hessian matrix obtained with the optim function in R.

### Model simulations: projections and scenario analyses

The parameter estimates were used to conduct 200 model simulations with different parameter sets to reflect uncertainty. Constant values were taken for the parameters estimated in step 1, and 200 samples of the parameters estimated in step 3. For the period up to 2 weeks before the analysis date, the means of the sampled contact matrices were used.

For the period after 2 weeks before the analysis date, additional uncertainty was added. Firstly, all 200 samples of each contact matrix were used because contact behaviour in that period was not yet adjusted by fits to the ICU data in step 3. Secondly, as no data were yet available to support potential future changepoints in transmissibility, we assumed that the transmissibility parameter remained constant, multiplied by random noise (range: 0.98–1.02) to reflect the uncertainty inherent in this assumption. The simulations resulted in 200 time courses of expected ICU admissions per day. To account for stochasticity in admissions, we used each once as mean of a Poisson distribution to simulate 200 stochastic outcomes per day. Finally, mean ICU admissions and 95% intervals for each day were plotted and presented as results.

### Model development during the pandemic

Up to 6 January 2021, the model and estimation procedure had undergone several changes, such as implementation of the SEEIIR compartments (instead of susceptible-infected-removed/recovered) and of age-dependent susceptibility and infectivity. Also, alternatives had been explored but not implemented, such as a negative binomial likelihood and a Markov chain Monte Carlo (MCMC) procedure to fit the model to data, which was too time-consuming for weekly fits. Changes to the model were carefully checked in sensitivity analyses when implemented (e.g. the transition from the continuous-time to discrete-time model, see Supplement), but not systematically challenged thereafter unless evidence became available to do so.

## Results

Towards the end of the first COVID-19 wave in April 2020, lengths of stay in hospital and ICU decreased and then remained constant throughout the rest of the study period. For instance, for patients aged 60–69 years in ICU, it took on average 11 days between infection and admission to the hospital general ward (Figure 1D), then an average of 2 days to be transferred to the ICU (Figure 1F), where they stayed on average 15 days, with 10% of patients staying longer than 34 days (Figure 1E). After discharge from the ICU, they stayed on average a further 10 days in a general ward before being discharged. Results for all ages and how these changes during 2020 can be found in the Supplement.

To assess the performance of the estimation procedure for the contact matrices and the transmissibility parameter *β*(*t*), we summarised the contact matrices by their associated reduction in transmission rates before fitting to ICU admission, *ϕ_T_*(0) and after fitting to ICU admissions, *ϕ_T_*(*t*) (Formula 3, Figure 2A). The numerical results can be seen in the Supplement. The reduction in transmission rates before fitting describes the effects of the control measures according to the estimated contact matrices. There was a large drop in transmissions around 18 March, when the first nationwide lockdown started. Transmissions then gradually increased until September 2020, when more measures were imposed until reaching a new minimum just before the Christmas holidays of 2020.

The reduction in transmission rates after fitting to the ICU admissions combines the estimated transmission matrices and the stepwise constant transmissibility function *β*(*t*). In this function, a total of six changepoints were needed as of 6 January 2021. The first was placed at the start of lockdown (18 March 2020) and the second about one week later. These time points reflect stepwise implementation of control measures and suggested a short-lasting extreme reduction in contact frequency before settling to a level approximately matching the contact matrix estimate. The estimates of the exact timing and magnitude of this short decrease in March 2020 changed several times during the year when the model changed, but that did not affect projections as shown in the Supplement. No further changepoints were needed until the end of September, after which three were needed during October and November in which infections were rising rapidly, but with only minor changes in transmissibility. A large correction was needed mid-November at *u*_11_ (Figure 2A), when transmissibility was estimated much higher than anticipated based on the control measures only.

The resulting 3-week projections during the first year of COVID-19 modelling showed that most were accurate in the sense that the actual (i.e. later observed) number of ICU admissions fell within the 95% prediction intervals (Figure 2B). The projections of the ICU admissions were too high at the end of March 2020, i.e. during the first peak, and at some instances during August 2020; projections were occasionally too low in November 2020. These projections improved when new data became available, providing evidence for new changepoints. As can be seen in the Supplement, these projections improved when new data became available, which provided evidence for new changepoints.

## Discussion

During the COVID-19 epidemic in the Netherlands, real-time short-term projections of COVID-19 ICU admissions were provided to the Dutch policymakers. Every week, the most recent data were incrementally incorporated into an age-structured transmission model in three steps. Firstly, transition probabilities and delay distributions for the clinical progression model were estimated, as well as most parameters of the transmission model. Secondly, contact matrices were estimated for each set of control measures. Thirdly, a time-varying transmissibility parameter was estimated that provided the best fit to the observed daily numbers of ICU admissions. This data assimilation process avoided a combinatorial explosion of parameter inferences for a model that increased in complexity over time, while minimising arbitrary decisions about the impact of control. The resulting model is semi-mechanistic: it combines statistical flexibility for an accurate and parsimonious description of the incidence of ICU admission in real time with a causal interpretation of the changes in contact matrices that allowed the anticipation of effects of recent and planned changes in control measures that are not yet detectable in the incoming data. The model complies with the theoretical (and practical) requirement that the dynamical behaviour of the model explains the observed dynamics, and, more importantly, the projections match the actual ICU admissions remarkably well.

A substantial change in the transmissibility parameter was estimated at only two points in the study period to correct the epidemic growth rate implied by the contact matrices (between *u*_1_ and *u*_2_, and at *u*_11_). The first of these corrections was for March 2020, in the first week of the first lockdown, when the estimated transmissibility parameter was lower than anticipated for 1 week before increasing to the anticipated level. A similar brief drop in the contact rates in this period in the Netherlands was reported by van Wees et al. [30]. The estimates likely reflect an actual behavioural change in the population in response to the rapidly rising number of daily hospitalisations with COVID-19. The second correction was for November 2020, during the second epidemic wave in the Netherlands, when estimated transmissibility parameter was higher than anticipated. This may, in part, be explained by a decreasing adherence of the population to control measures. Another explanation is a seasonal change in transmissibility, with higher values in winter. Indeed, after implementing a seasonally dependent transmission parameter into the model, implemented in January 2021, subsequent to our study period, a much smaller correction was needed. Naturally, short-term projection intervals did not capture the actual reported numbers of ICU admissions at these two time points, with projections improving shortly thereafter, when new data became available. In addition, some projection interval failed to capture admission in August, when the numbers of daily ICU admissions were low. Overall, the projections captured the later reported dynamics in ICU admissions reasonably well, which is a prerequisite for informing policymakers.

In practice, the need for timely short-term projections required making a number of assumptions, the impact of which should be considered. The transmission model used here is deterministic and assumes homogeneous mixing within age groups for the whole Dutch population. Even though the model did not capture spatial heterogeneity and local stochastic effects, the simulations captured the national average well for a population of 17 million persons. Many parameters and delay distributions were estimated independently, which risks underestimating uncertainty and ignoring potential correlation between parameter values. However, it seems that less accurate projections were generally caused by differences in anticipated contact rates – later corrected by estimates of the time-dependent transmissibility, as explained above – and not by overly narrow prediction intervals resulting from the use of independent estimates. By estimating most parameters in the first step, the inferential procedure was time-efficient, ensuring sufficient time to update all weekly estimates and to integrate the most recent data into the transmission model. This efficiency proved vital when more complex extensions of the model were deployed later in the pandemic. Estimation of the contact matrices was done by combining contact data from before the pandemic and reductions estimated by two researchers in a standardised protocol. This was feasible given the time constraints and turned out well. A major uncertainty in this protocol was future adherence to control measures. This may be inferred from behavioural surveys, but more research is needed to evaluate this possibility for real-time prediction.

## Conclusions

This study has focused on the epidemic dynamics over the period February 2020 to January 2021 a period when the vast majority of the population was susceptible to infection and recurrent waves were a direct consequence of contact behaviour. Because the model incorporates basic principles dictated by epidemic theory, it had potential for use in the range of epidemiological situations that occurred since then. Firstly, the model can be readily adapted to include compartments and stratified parameters for vaccinated persons such that it could be used to make projections in a situation with COVID-19 vaccination. Secondly, the model can be adapted to include dynamic transmissibility and immune escape rates, when data on genomic surveillance provide evidence thereof, caused by variants of the virus. Thirdly, the loss of immunity can be modelled to facilitate the description of endemic dynamics of an infection spreading in a population that has been vaccinated or previously infected. When the epidemiological situation becomes more complex, when more data sources are available, and when research and policy questions become more interrelated, the approach of relating the transmission model to incoming data proves efficient and flexible. We believe this testifies to its practical merits.

## Supplementary Data

6086000 Supplementary Material

## References

[1] Fountoulakis KN Karakatsoulis G Abraham S Adorjan K Ahmed HU Alarcón RD Results of the COVID-19 mental health international for the general population (COMET-G) study. Eur Neuropsychopharmacol. 2022;54:21-40. 10.1016/j.euroneuro.2021.10.004 34758422 PMC8609892

[2] McDonald SA Lagerweij GR de Boer P de Melker HE Pijnacker R Mughini Gras L The estimated disease burden of acute COVID-19 in the Netherlands in 2020, in disability-adjusted life-years. Eur J Epidemiol. 2022;37(10):1035-47. 10.1007/s10654-022-00895-0 35951278 PMC9366822

[3] Ainslie KEC Backer JA de Boer PT van Hoek AJ Klinkenberg D Korthals Altes H A scenario modelling analysis to anticipate the impact of COVID-19 vaccination in adolescents and children on disease outcomes in the Netherlands, summer 2021. Euro Surveill. 2022;27(44):2101090. 10.2807/1560-7917.ES.2022.27.44.2101090 36330824 PMC9635025

[4] Ferretti L Wymant C Kendall M Zhao L Nurtay A Abeler-Dörner L Quantifying SARS-CoV-2 transmission suggests epidemic control with digital contact tracing. Science. 2020;368(6491):eabb6936. 10.1126/science.abb6936 32234805 PMC7164555

[5] Birrell P Blake J van Leeuwen E Gent N De Angelis D. Real-time nowcasting and forecasting of COVID-19 dynamics in England: the first wave. Philos Trans R Soc Lond B Biol Sci. 2021;376(1829):20200279. 10.1098/rstb.2020.0279 34053254 PMC8165585

[6] Dong E Du H Gardner L. An interactive web-based dashboard to track COVID-19 in real time. Lancet Infect Dis. 2020;20(5):533-4. 10.1016/S1473-3099(20)30120-1 32087114 PMC7159018

[7] Campillo-Funollet E Van Yperen J Allman P Bell M Beresford W Clay J Predicting and forecasting the impact of local outbreaks of COVID-19: use of SEIR-D quantitative epidemiological modelling for healthcare demand and capacity. Int J Epidemiol. 2021;50(4):1103-13. 10.1093/ije/dyab106 34244764 PMC8407866

[8] Cramer EY Ray EL Lopez VK Bracher J Brennen A Castro Rivadeneira AJ Evaluation of individual and ensemble probabilistic forecasts of COVID-19 mortality in the United States. Proc Natl Acad Sci USA. 2022;119(15):e2113561119. 10.1073/pnas.2113561119 35394862 PMC9169655

[9] Dehning J Zierenberg J Spitzner FP Wibral M Neto JP Wilczek M Inferring change points in the spread of COVID-19 reveals the effectiveness of interventions. Science. 2020;369(6500):eabb9789. 10.1126/science.abb9789 32414780 PMC7239331

[10] Krymova E Béjar B Thanou D Sun T Manetti E Lee G Trend estimation and short-term forecasting of COVID-19 cases and deaths worldwide. Proc Natl Acad Sci USA. 2022;119(32):e2112656119. 10.1073/pnas.2112656119 35921436 PMC9371653

[11] Mullah MAS Yan P. A semi-parametric mixed model for short-term projection of daily COVID-19 incidence in Canada. Epidemics. 2022;38:100537. 10.1016/j.epidem.2022.100537 35078118 PMC8767942

[12] Muñoz-Organero M Queipo-Álvarez P. P. Deep spatiotemporal model for COVID-19 forecasting. Sensors (Basel). 2022;22(9):3519. 10.3390/s22093519 35591208 PMC9101138

[13] Paireau J Andronico A Hozé N Layan M Crépey P Roumagnac A An ensemble model based on early predictors to forecast COVID-19 health care demand in France. Proc Natl Acad Sci USA. 2022;119(18):e2103302119. 10.1073/pnas.2103302119 35476520 PMC9170016

[14] Panaggio MJ Rainwater-Lovett K Nicholas PJ Fang M Bang H Freeman J Gecko: A time-series model for COVID-19 hospital admission forecasting. Epidemics. 2022;39:100580. 10.1016/j.epidem.2022.100580 35636313 PMC9124631

[15] Bekker R Uit Het Broek M Koole G. Modeling COVID-19 hospital admissions and occupancy in the Netherlands. Eur J Oper Res. 2023;304(1):207-18. 10.1016/j.ejor.2021.12.044 35013638 PMC8730382

[16] Van Wees J-D, Osinga S, Van der Kuip M, Tanck M, Hanegraaf M, Pluymaekers M, et al. Forecasting hospitalization and ICU rates of the COVID-19 outbreak: an efficient SEIR model2020 7 March 2023. Available from: https://www.researchgate.net/publication/340286949.

[17] Verberk JDM Vos RA Mollema L van Vliet J van Weert JWM de Melker HE Third national biobank for population-based seroprevalence studies in the Netherlands, including the Caribbean Netherlands. BMC Infect Dis. 2019;19(1):470. 10.1186/s12879-019-4019-y 31138148 PMC6537387

[18] Verelst F Hermans L Vercruysse S Gimma A Coletti P Backer JA SOCRATES-CoMix: a platform for timely and open-source contact mixing data during and in between COVID-19 surges and interventions in over 20 European countries. BMC Med. 2021;19(1):254. 10.1186/s12916-021-02133-y 34583683 PMC8478607

[19] Backer JA Bogaardt L Beutels P Coletti P Edmunds WJ Gimma A Dynamics of non-household contacts during the COVID-19 pandemic in 2020 and 2021 in the Netherlands. Sci Rep. 2023;13(1):5166. 10.1038/s41598-023-32031-7 36997550 PMC10060924

[20] Vos ERA den Hartog G Schepp RM Kaaijk P van Vliet J Helm K Nationwide seroprevalence of SARS-CoV-2 and identification of risk factors in the general population of the Netherlands during the first epidemic wave. J Epidemiol Community Health. 2020;75(6):489-95. 10.1136/jech-2020-215678 33249407 PMC8142429

[21] Vos ERA van Boven M den Hartog G Backer JA Klinkenberg D van Hagen CCE Associations between measures of social distancing and severe acute respiratory syndrome coronavirus 2 seropositivity: a nationwide population-based study in the Netherlands. Clin Infect Dis. 2021;73(12):2318-21. 10.1093/cid/ciab264 33772265 PMC8083720

[22] Ward M Brandsema P van Straten E Bosman A. Electronic reporting improves timeliness and completeness of infectious disease notification, The Netherlands, 2003. Euro Surveill. 2005;10(1):7-8. 10.2807/esm.10.01.00513-en 29183539

[23] Dongelmans DA Termorshuizen F Brinkman S Bakhshi-Raiez F Arbous MS de Lange DW Characteristics and outcome of COVID-19 patients admitted to the ICU: a nationwide cohort study on the comparison between the first and the consecutive upsurges of the second wave of the COVID-19 pandemic in the Netherlands. Ann Intensive Care. 2022;12(1):5. 10.1186/s13613-021-00978-3 35024981 PMC8755895

[24] Diekmann O, Heesterbeek H, Britton T. Mathematical tools for understanding infectious disease dynamics. Princeton: Princeton University Press; 2013. p. 502.

[25] Bergstrom CT, West JD. Calling bullshit: the art of skepticism in a data-driven world. New York: Penguin Random House; 2020. p. 318.

[26] Wikipedia. Fermi Problem: Wikipedia. [Accessed: 27 Feb 2024]. Available from: https://en.wikipedia.org/wiki/Fermi_problem

[27] van de Kassteele J Van Eijkeren J Wallinga J. Efficient estimation of age-specific social contact rates between men and women. Ann Appl Stat. 2017;11(1):320-39. 10.1214/16-AOAS1006

[28] R Core Team. R: A language and environment for statistical computing. Vienna: R Foundation for Statistical Computing; 2020. Available from: https://www.R-project.org

[29] Burnham KPA, Anderson DR. Model selection and multimodel inference. A practical information-theoretic approach. New York: Springer; 2002. p. 488.

[30] van Wees J-D Van Der Kuip M Osinga S Van Westerloo D Tanck M Hanegraaf M Performance of progressive and adaptive COVID-19 exit strategies: a stress test analysis for managing intensive care unit rates. medRxiv. 2020 . 10.1101/2020.05.16.20102947

[31] Backer JA Klinkenberg D Wallinga J. Incubation period of 2019 novel coronavirus (2019-nCoV) infections among travellers from Wuhan, China, 20-28 January 2020. Euro Surveill. 2020;25(5):2000062. 10.2807/1560-7917.ES.2020.25.5.2000062 32046819 PMC7014672

[32] Lauer SA Grantz KH Bi Q Jones FK Zheng Q Meredith HR The incubation period of coronavirus disease 2019 (COVID-19) from publicly reported confirmed cases: estimation and application. Ann Intern Med. 2020;172(9):577-82. 10.7326/M20-0504 32150748 PMC7081172

[33] Ganyani T Kremer C Chen D Torneri A Faes C Wallinga J Estimating the generation interval for coronavirus disease (COVID-19) based on symptom onset data, March 2020. Euro Surveill. 2020;25(17):2000257. 10.2807/1560-7917.ES.2020.25.17.2000257 32372755 PMC7201952

[34] Tindale LC Stockdale JE Coombe M Garlock ES Lau WYV Saraswat M Evidence for transmission of COVID-19 prior to symptom onset. eLife. 2020;9:e57149. 10.7554/eLife.57149 32568070 PMC7386904

